# Third-generation cryotherapy reduces time to surgery and local complications in patients with ankle fractures: a prospective randomised controlled trial

**DOI:** 10.1186/s13018-025-06599-x

**Published:** 2026-03-14

**Authors:** Riccardo Maria Lanzetti, Alessio Giai Via, Francesco Anzano, Gennaro Pipino, Raffaella Alonzo, Carmelo D’Arrigo, Stefania De Sanctis, Marco Spoliti, Giovanna Fidone, Filippo Migliorini, Nicola Maffulli

**Affiliations:** 1 Department of Orthopaedic and Trauma Surgery, A.O. San Camillo-Forlanini, Rome, Italy; 2 Department of Orthopaedic and Trauma Surgery, San Raffaele Hospital, University San Raffaele, Milan, Italy; 3 Department of Orthopaedic and Trauma Surgery Villa Erbosa Hospital, Bologna, Italy; 4 Department of Orthopaedic and Trauma Surgery, San Paolo Hospital, Civitavecchia, Italy; 5 Department of Orthopaedic and Trauma Surgery, Academic Hospital of Bolzano (SABES-ASDAA), via Lorenz Böhler 5, Bolzano, 39100 Italy; 6 Department of Life Sciences, Health, and Health Professions, Link Campus University, Via del Casale di San Pio V, Rome, 00165 Italy; 7 Orthopaedic Unit, S. Andrea Hospital, University of Rome “La Sapienza”, Via di Grottarossa 1035, Rome, Italy; 8 Queen Mary’s University London, Mile End Hospital, Mile End Road, London, E1 4NS UK; 9 Department of Trauma and Reconstructive Surgery, University Hospital of Halle, Martin-Luther University Halle-Wittenberg, Ernst-Grube-Street 40, 06097 Halle (Saale), Germany

**Keywords:** Cryotherapy, Ankle fractures, Ankle surgery, Time to surgery

## Abstract

**Background:**

Ankle fractures are common, and cryotherapy is routinely used to reduce pain, swelling and local skin complications, both before and after surgery. The aim of this study is to report the results with the use of pre-operative third-generation cryotherapy (Z-One^®^, Zamar, Italy) in the management of patients with ankle fractures. We investigated the time to surgery, pain, opioid intake, and local skin complications.

**Methods:**

169 patients with ankle fracture were randomised into two groups, the cryotherapy group (89 patients) and the control group (C: 80 patients). The time-to-surgery, Visual Analogue Scale (VAS) and the analgesic drug demands (Morphine Sulfate 10 mg/ml solution for injection) were recorded. The development of skin complications was assessed on a daily basis. BMI and the number of cigarettes smoked were also recorded.

**Results:**

The mean time-to-surgery was shorter in patients treated with cryotherapy compared to the control group (34.78 h vs. 91.44 h, *p* < 0.001). Significant differences between treatments and controls were found for VAS, morphine intake (number of vials), and skin complications. The mean preoperatory VAS and morphine consumption were lower in the cryotherapy group compared to controls (mean VAS 2.04 vs. 5.9, mean morphine consumption 0.1 mg vs. 0.83 mg). In the cryotherapy group, 4.5% of patients developed a skin complication compared to 28.7% of the control group; 85% of skin-related problems occurred in the non-cryotherapy group (*p* < 0.001).

**Conclusion:**

Preoperative third-generation cryotherapy is effective in reducing time to surgery, preoperative pain, and opioid intake in patients hospitalised for ankle fractures. It is also effective in reducing the occurrence of skin complications. No major complications related to the use of the device were reported. Third-generation cryotherapy is useful in the perioperative management of patients necessitating surgery for ankle fractures.

*Clinical Trial Registration *NCT06396364.

*Level of evidence *I (RCT).

## Introduction

Ankle fractures are common, accounting for about 10% of all fractures [1]. They are common in young and active patients, with a reported prevalence of 15%-25% of all sports-related injuries [2], but their incidence is also increasing in elderly patients, being the third most common fracture after the 6th decade of life [3]. Wound complications such as dehiscence and surgical site infections are frequent local complications, leading to poor functional outcomes and increasing healthcare costs by more than 300% [4]. Local complications may be related to the time to surgery, although the literature is not univocal.

Cryotherapy is routinely used to reduce pain and swelling both before and after surgery [5]. It is commonly used to manage pain and swelling in several musculoskeletal disorders [6, 7], as well as an adjuvant treatment in systemic disorders [8]. Cryotherapy acts by decreasing blood flow, reducing painful stimuli, and preventing local inflammation; in addition, it has antiproliferative and chondroprotective effects [8].

Conventional cryotherapy consists of ice gels or packages that are directly applied to the area of pain and soreness. Second-generation cryotherapy involves the use of cuff devices, with iced water flowing through, on which constant pressure can be applied [9]. The increased external pressure reduces the pressure gradient between blood vessels and tissues, and discourages leakage from capillaries. A common problem is the development of reactive vasodilation, consequent to the rapid increase in the local temperature after the procedure. Third-generation devices deliver water-free computerised cryotherapy, which maintains a constant temperature through different anatomical layers, while allowing progressive changes in temperature and pressure to avoid reactive vasodilation [10]. Independent of the location and the type of device used, cryotherapy is generally well tolerated and increases patient satisfaction [9, 10].

Although the use of cryotherapy is traditionally believed to reduce pain, swelling, local skin complications, and the need for analgesia, its effects on ankle surgery are contradictory. Furthermore, no studies on third-generation devices have been conducted on ankle fractures.

We report the results of using third-generation cryotherapy (Z-One^®^, Zamar, Italy) in the preoperative management of ankle fractures. The primary aim was to assess the time to surgery. We investigated treatment efficacy based on reported pain, morphine consumption, and preoperative local skin complications. The safety and tolerability of the procedure were also studied. The working hypothesis is that third-generation cryotherapy reduces time to surgery and is useful in the surgical management of ankle fractures.

## Materials and methods

**Fig. 1:**
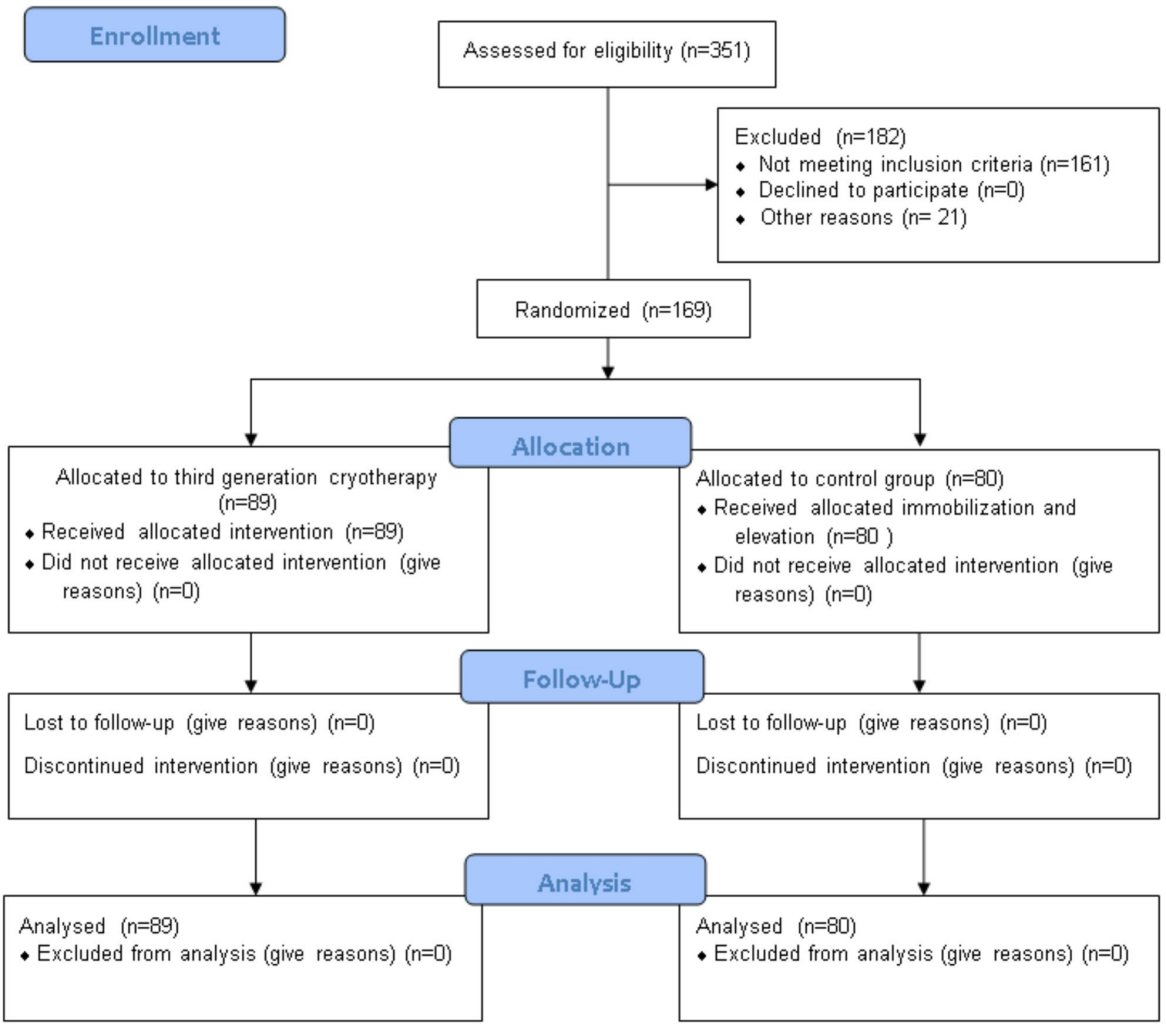
Study flow diagram

We analysed the prospectively collected data of 169 patients with ankle fractures, treated with open reduction and internal fixation (ORIF). Patients were randomly grouped into two groups in the Emergency Department at the time of diagnosis. The cryotherapy group (89 patients) were treated with a third-generation cryotherapy device (Z-One^®^, Zamar), which was applied daily for two hours two times a day up to the day of surgery. The leg was immobilised in a walking boot, which was removed when the cryotherapy device was applied. The control group (80 patients) did not use cryotherapy before surgery, the leg was immobilised in a below-knee back slabs, and elevation of the injured limb was implemented. Patients were admitted into two hospitals (Azienda Ospedaliera San Camillo Forlanini-Roma and Ospedale San Paolo–Civitavecchia) between 2021 and 2023 (NCT06396364). All patients had signed a written consent, and the study was approved by the local ethics committee. The details of the CONSORT statement allocation are reported in Fig. 1.

### Inclusion and exclusion criteria

Patients admitted to the Emergency Department with an ankle fracture were included in the current study. Exclusion criteria were open fractures, fracture-dislocations which required external fixation, patients with one or more associated fractures and polytrauma patients. Patients who were not able to comply with the pre- and post-surgical indications were also excluded.

### Patients’ assessment and randomisation

A detailed physical examination was conducted in all patients by local orthopedic surgeons. All patients underwent standard radiographs to diagnose the fracture, and computed tomography for preoperative planning if indicated. After the diagnosis was made and surgical treatment was indicated, patients were admitted to the Department of Orthopedic and Trauma Surgery. Patients were randomised into two groups (cryotherapy and control group) by blocked randomisation with random block sizes to ensure that the patients were balanced. The randomisation of participants was conducted by the authors G.F. and R.A., who didn’t operate on the participants. Other authors (R.M.L, C.D.A., R.A.) opened the envelopes, assessed the preoperative local skin condition, assessed a positive wrinkle sign and determined that patients were eligible for surgery. Operating surgeons (C.D.A., M.S., A.G.V.) were blinded to the group allocation. Other Authors validated the outcomes.

**Fig. 2:**
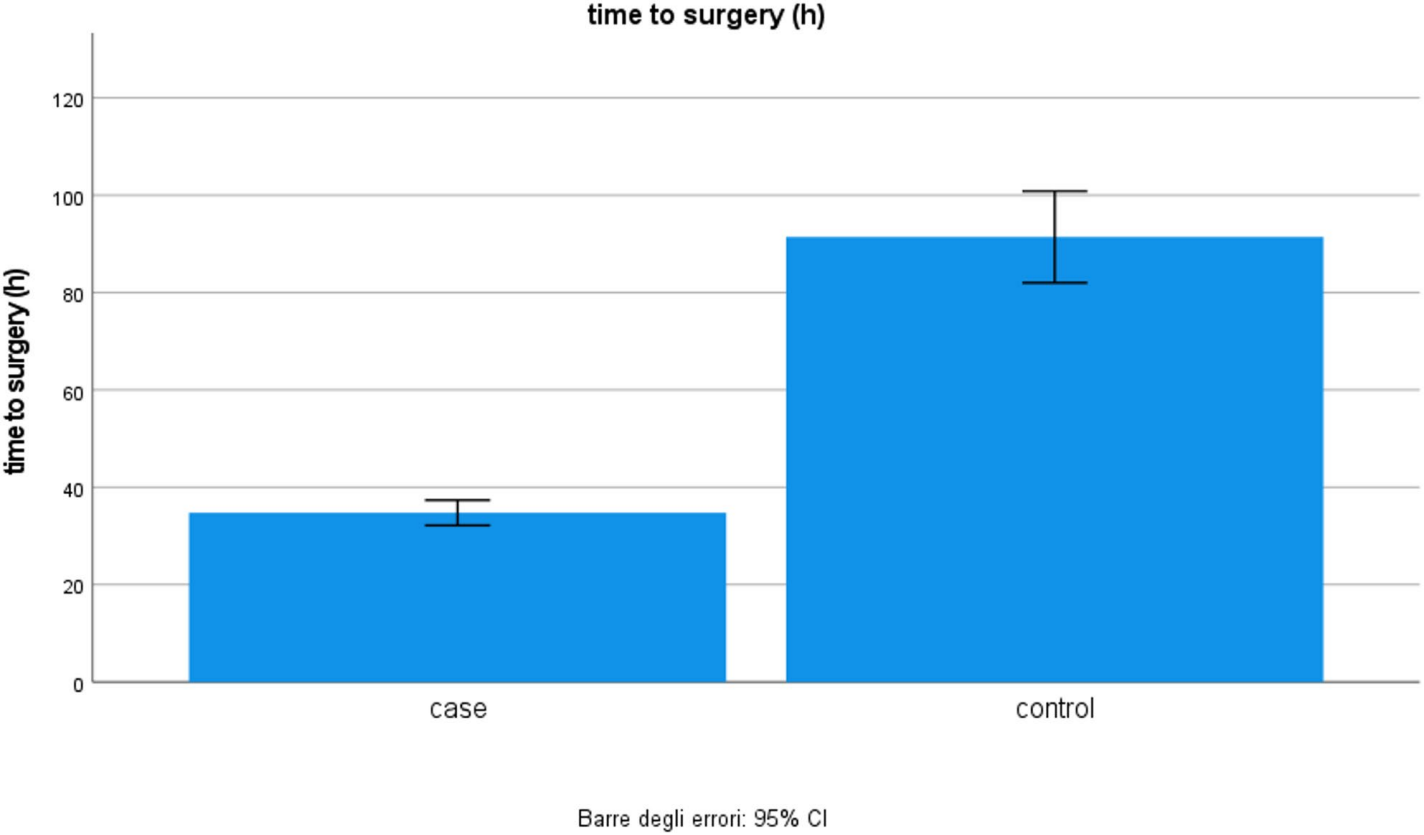
Time to surgery. The time to surgery is significantly shorter in patients treated with the third cryotherapy device compared to the control group

Sample size calculations were conducted using the G-power program (IBM Inc., Armonk, NY, USA). Patients in the cryotherapy group were immobilised in a walking boot to allow the use of the cryotherapy device (Fig. 2). Patients in the control group were immobilised in below-knee back slabs. Pain was evaluated according to the Visual Analogue Scale (VAS) and analgesic drug consumption (mg of morphine). The Body Mass Index (BMI), number of cigarettes smoked, and presence of preoperative skin complications were assessed.

### Surgical technique

The wrinkle sign was chosen as the clinical criteria to plan surgery. As the swelling receded and the skin began to wrinkle both on the lateral and medial sides, the patient was eligible for surgery. Under loco-regional anesthesia, the patient was positioned supine with a sandbag under the ipsilateral buttock, or prone if a posterolateral surgical approach was indicated. A tourniquet was applied at the thigh, the lower limb was prepared and draped in the usual sterile fashion, exsanguinated, and the tourniquet was inflated. The lateral malleolus was approached first, reduced and fixed with a plate and screws. Then, the surgical technique varied depending on the fracture pattern, the presence of syndesmosis injury and associated soft tissue injury. The skin was sutured with non-absorbable sutures, and the ankle was immobilised in a walking boot. All patients received intraoperative antibiotic treatment, intravenous controlled analgesia 24 to 48 h after surgery, and standard thromboembolic prophylaxis with low molecular weight heparin (LMWE) for up to complete weight bearing. The ankle was immobilised in a walking boot, but passive ankle motion was encouraged from the first post-operative day.

### Statistical analysis

An a priori power analysis was performed using preliminary data based on the time-to-surgery variable. The initial estimate suggested a minimum of 4 units per group to achieve 90% power. Subsequent analysis of the full dataset indicated higher variability; under these conditions, the minimum required sample size per group would be 8. Univariate descriptive analysis of the variables under study was carried out by calculating the centrality and variability indices for the quantitative variables and frequency tables for the variables.

The homogeneity between the cryotherapy group and control group for the variables sex, age, type of fracture, diabetes, hypercholesterolemia, venous insufficiency, and BMI class was checked. Depending on the nature of the variables, a t-test or a chi-square test was used.

Any significant differences between the cryotherapy and control groups were assessed using independent samples t-tests for quantitative variables and chi-square tests for qualitative variables. Where the sample size was not sufficient, the non-parametric Mann-Whitney test for independent samples was used.

An alpha significance level of 0.05 was used in all analyses mentioned. For the statistical analysis of data, IBM SPSS Statistics software version 28 was used.

## Results

Basic characteristics of the study population are reported in Table 1.

From the power analysis, the minimum sample size to obtain a statistical power of at least 0.9 with a significance level of 0.05 is at least four units for each group. Specifically, the power reached is 0.978.

The chi-square test showed no association between belonging to a group and the variables sex, type of fracture, diabetes, hypercholesterolemia, chronic venous insufficiency, and BMI. Therefore, the groups were homogeneous concerning these variables. The mean age of the cryotherapy group was 50.63 years (SD 16,271; Std. Error Mean 1.725), and the mean age of the control group was 53.25 (SD 14,541; Std. Error Mean 1.626), with no significant differences.

The mean time to surgery was shorter in the cryotherapy group. The mean time to surgery in the cryotherapy group was 34.78 h. (median 36.0 h., S.D. 12.31, Mean Std. Error 1.30). In comparison, in controls, it was 91.44 h. (median 78.5 h., S.D. 42.25, Mean Std. Error 4.72) (Fig. 2). The t-test was performed according to the different subtypes of fractures. Given the low sample size of the two groups, the non-parametric Mann-Whitney test was used. Third-generation cryotherapy significantly decreases time to surgery independently of the type of fracture (Table II).

A significant difference between the groups was also investigated for the secondary outcomes: VAS, morphine consumption (mg), and skin complications.

**Fig. 3:**
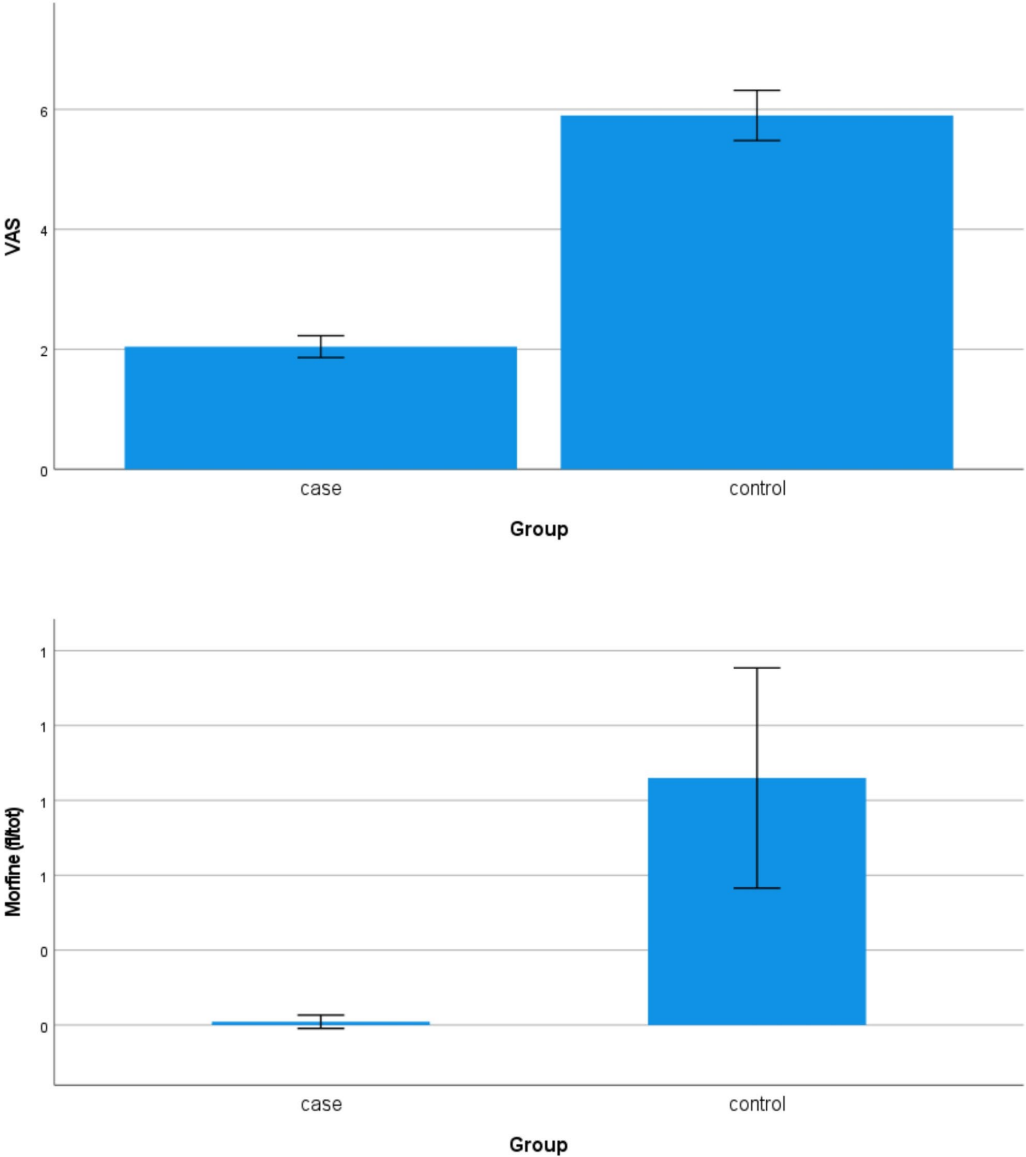
The mean pre-operatory VAS (**A**) and morphine consumption (Mg of morphine—**B**) were significantly lower (*p*-value < 0.001) in the cryotherapy group compared to the control group

The mean preoperative VAS and morphine consumption were lower in the cryotherapy group compared to controls. (mean VAS 2.04, S.D. 0.865, Mean Std. Error 0.092, and 5.9, S.D. 1.88, Mean Std. Error 0.210 respectively; mean morphine assumption 0.1 mg, S.D. 0.106, Mean Std. Error 0.011, and 8.3 mg, S.D. 1.652, Mean Std. Error 0.185, respectively) Fig. 3.

**Table 1:** Basic characteristics of the study population. (*Fracture type: ankle fractures were classified according to the Weber and Lauge-Hansen Classification)

		Group			
		Cryotherapy		Control	
		Count	%	Count	%
Sex	M	56	62.9%	45	56,3%
	F	33	37.1%	35	43,8%
Fracture type *	Trimalleolar fracture	26	29.2%	19	23,8%
	Bimalleolar fracture	29	32.6%	36	45,0%
	Tibial malleolus only	0	0.0%	0	0,0%
	Malleolar fracture only	17	19.1%	15	18,8%
	Trimalleolar fracture with syndesmotic disruption	17	19.1%	10	12,5%
Skin injuries	No	85	95.5%	57	71.3%
	Yes	4	4.5%	23	28.7%
Diabetes	No	73	82.0%	70	87.5%
	Yes	16	18.0%	10	12.5%
Hypercholesterolemia	No	73	82.0%	67	83.8%
	Yes	16	18.0%	13	16.3%
Venous insufficiency	No	82	92.1%	76	95.0%
	Yes	7	7.9%	4	5.0%
BMI	Underweight	0	0.0%	0	0.0%
	Normal	42	47.2%	46	57.5%
	Overweight	35	39.3%	23	28.7%
	Obesity I stage	12	13.5%	7	8.8%
	Obesity II stage	0	0.0%	3	3.8%
	Obesity III stage	0	0.0%	1	1.3%

**Table 2:** Cryotherapy reduces the time to surgery for all types of ankle fractures compared to controls

		Group						*p*-value°
		Cryotherapy			Control			
		Time to surgery (h)			Time to surgery (h)			
		Mean	Median	Standard Deviation	Mean	Median	Standard Deviation	
Type of fracture	Trimalleolar	36.23	39.00	8.66	102.26	90.00	42.73	< 0.001
	Bimalleolar	33.79	30.00	11.94	79.94	75.00	24.60	< 0.001
	Medial malleolus	.	.	.	.	.	.	
	Lateral malleolus	31.65	24.00	11.32	93.33	72.00	63.43	< 0.001
	Trimalleolar with TPA dislocation	37.35	28.00	17.79	109.40	111.50	47.61	< 0.001

**Table 3:** Cutaneous complications crosstabulation

Cutaneous Complications Crosstabulation					
			Skin lesions		Total
			No	Yes	
Group	Cryotherapy	Count	85	4	89
		% within Group	95.5%	4.5%	100.0%
		% within skin lesions	59.9%	14.8%	52.7%
	Controls	Count	57	23	80
		% within Group	71.3%	28.7%	100.0%
		% within skin lesions	40.1%	85.2%	47.3%
Total		Count	142	27	169
		% within Group	84.0%	16.0%	100.0%
		% within skin lesions	100.0%	100.0%	100.0%

The chi-square test was used to investigate the development of preoperative skin complications. We recorded four skin complications in the cryotherapy group (4 serous blisters; 4/89, 4.5%), and 23 skin lesions in the control group (6 severe edema, 13 serous blisters, 4 hemorrhagic blisters; 23/80; 28.7%). 85.2% of skin complications occurred in the control group (Table III). The difference was statistically significant (p-value < 0.001). No side effects were reported in the cryotherapy group.

## Discussion

Ankle fractures are frequent, with an overall incidence rate ranging from 45 to 107/1.000 person-years, accounting for about 10% of all fractures [11, 12]. They are more common in men below 50 years of age, then females became predominant and the age-specific incidence rates decreased in both sexes [11, 12]. The incidence of ankle fractures has increased by up to 300% in the last 30 years, especially in the elderly population. Unfortunately, complications following surgical treatment are common, ranging from 10% to 30% of cases, with rates increasing to 40% in the elderly [4, 13]. Deep infections represent the most serious complications, increasing the risk of poor functional outcome, and may increase the healthcare costs by more than 300% [4, 14]. Time-to-surgery may influence the development of local skin complications [15], although information about the influence of delayed surgery is still ambiguous.

Cryotherapy decreases swelling, joint effusion, and relieves pain. It induces local vasoconstriction, reduces capillary leakage, and diminishes blood flow to the joint and soft tissues [8]. It can moderate the inflammatory cascade by decreasing pro-inflammatory cytokines (IL-6, IL-1, VEGF, PG-E2, NF-kB-p65) in the synovial fluid, and increases the synthesis of pyruvate, which has an antioxidant effect [16, 17]. It also decreases the electrical conduction speed through nerves and limits transmission of the electrical impulses to the sensory neurons in the posterior horn of the medullary canal, thus reducing pain [15]. In vitro cryotherapy regulates the intracellular pH, which reduces muscular fatigue and increases the synthesis of alanine, which has a chondroprotective effect [16, 18]. Finally, it decreases the proliferation rate of cells, including bacteria, hence it may prevent wound infection in foot and ankle surgery [6].

Independent of the location and the type of device used, cryotherapy is generally well tolerated and increases patient satisfaction [7, 10, 19–21]. Conventionally, cryotherapy is delivered through ice packs, which have been proven effective in reducing edema and pain in ankle fractures. A common burden of ice pack cryotherapy is the development of reactive vasodilation, consequent to the rapid increase in the local temperature after the procedure. Seemingly, the risk of frostbite in the ankle and foot region is extremely rare, but its consequences might be devastating, requiring active surveillance and prompt treatment [22, 23].

Second-generation cryotherapy involves the use of cuff devices, with iced water flowing through, on which constant pressure can be applied [24]. The increased external pressure reduces the pressure gradient between blood vessels and tissues, and discourages leakage from capillaries. Thanks to the effect of compression, these devices are effective in reducing edema and ankle swelling after trauma. Compressive cryotherapy is well tolerated and efficacious in decreasing the use of analgesic after knee and ankle surgery, and it is becoming a standard of care in postoperative rehabilitation of patients with ankle fractures [25]. However, it does not decrease the development of postoperative complications, including infections [26]. No differences in pain reduction and ROM were reported using second-generation compared to first-generation devices following total knee arthroplasty [27]. Furthermore, second-generation cryotherapy devices are not able to maintain a constant cold temperature for a long time.

**Fig. 4:**
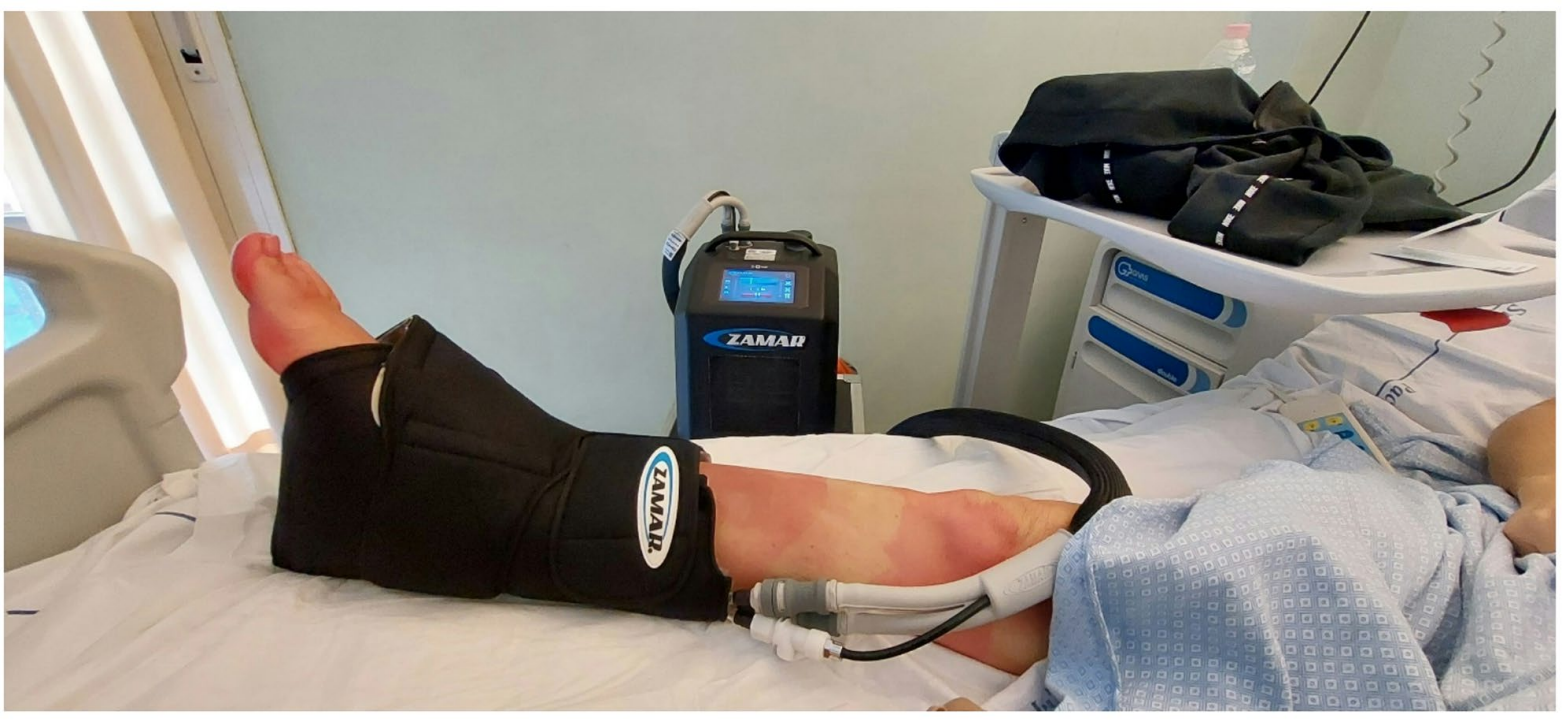
Third-generation cryotherapy device

To avoid these problems, third-generation devices use computed cryotherapy to deliver gradual changes in pressure and temperature. The software produces a progressive drop in temperature and a controlled, slow return to a room-temperature environment, avoiding reactive vasodilation (Fig. 4).

Despite the above-mentioned beneficial results of cryotherapy in the pre- and postoperative care of ankle surgery, the lack of standardised protocols and few level 1 studies make it difficult to appreciate its benefits. In addition, there’s scarce evidence on the benefits of the preoperative use of cryotherapy [14].

Third-generation cryotherapy is effective in reducing time-to-surgery, pain and wound complications. Mean VAS score and morphine consumption were significantly lower in the cryotherapy group compared to controls. The reduction of morphine intake reduces their dose-related side effects, and improves patient satisfaction [20, 28]. Cryotherapy significantly reduces the prevalence of preoperative skin complications, which are common in ankle trauma. The most common are local erythema, severe edema, blistering, and necrosis, which are predictors of negative outcomes [4]. Notably, 95.5% of the cryotherapy group did not show skin complications compared to 71.3% of the control group. When we considered all patients with skin injuries, 85.2% of these patients belonged to the control group. We hypothesise that the reduction of time to surgery is due to the faster reduction of swelling and a significant reduction of preoperative local skin complications. BMI, cigarette consumption and acetaminophen intake did not affect primary and secondary outcomes. Finally, no side effects such as deep venous thrombosis, frostbite, excessive pain, or compartmental syndrome were reported. We found only a recent retrospective study comparing the effect of ice pack and third-generation cryotherapy after unicompartmental knee arthroplasty surgery, reporting significant improvements in postoperative pain control and knee function [29]. However, the study’s retrospective design and lack of well-designed studies make further level I studies necessary to validate these promising results.

Our study certainly had some limitations. Firstly, there was no standardised protocol that could define the best timing and frequency of cryotherapy. The patients were not blinded about the treatment, because the cryotherapy group was immobilised in a walking boot, while the control group was immobilised in a back slab. However, the surgeon was blinded about the patients’ allocation because the immobilisation was removed before entering the surgical theatre. Thirdly, literature results on pre-operative cryotherapy are scarce, and some have reported a paradoxical increase in time-to-surgery with cryotherapy. Some patients might have taken other analgesics, including NSAIDs, which were not reported in the study. No investigation has been done on the total length of stay, as most of our patients are discharged the day after surgery, in functional outcomes, or in the development of postoperative complications (i.e. wound dehiscence and surgical site infection).

We are aware that a full health economics analysis has not been performed and was not planned. However, the favourable impact on pre-operative length of stay is likely to result in cost savings for our healthcare system.

## Conclusion

Third-generation cryotherapy is effective in reducing time-to-surgery, pre-operative pain and opioid intake in patients undergoing surgery for ankle fractures. It is also effective in reducing the occurrence of skin complications. The patients themselves administer it, and patient monitoring is not needed. It is safe, with no major complications related to its use. Third-generation cryotherapy may be useful in perioperative treatment of patients hospitalised for ankle fractures, as it may reduce the hospital stay. However, standardised protocols and further studies are required to confirm these encouraging results.

## Data Availability

All data generated or analysed during this study are included in thispublished article. The datasets used and/or analysed during the current study are also availablefrom the corresponding author on reasonable request.

## Abbreviations

ORIF: open reduction and internal fixation
VAS: Visual Analogue Scale
BMI: Body Mass Index
LMWE: Low molecular weight heparin
NSAIDs: Non-steroidal anti-inflammatory drugs
